# Effect of Brain and Pulse Waves on Safety Consciousness and Safety Commitment of Workers at Construction Sites

**DOI:** 10.3390/s21082753

**Published:** 2021-04-13

**Authors:** Young-Jun Park, Un-Na Lim, Sangwoo Park, Jae-Han Shin

**Affiliations:** 1 Department of Civil Engineering and Environmental Sciences, Korea Military Academy, Seoul KS013, Korea; parky@mnd.go.kr (Y.-J.P.); ptstwt@korea.ac.kr (S.P.); 2 Brain Based Coaching & Counseling Center, Seoul KS013, Korea; coffer00@ube.ac.kr; 3 Department of Brain Education, University of Brain Education, Cheonan KS002, Korea

**Keywords:** brain wave, pulse wave, safety consciousness, safety commitment, construction site

## Abstract

Even though individual mental and health status largely affects the safety in industrial sites, most studies for preventing industrial accidents are mainly focused on external factors such as regulations, education, etc. In this study, the effect of individual factors on safety (i.e., safety consciousness and safety commitment) was analyzed by collecting brainwave and pulse data at construction sites where industrial accidents have occurred with the highest percentage. The effects of brain stress, concentration, brain activity, and left and right brain imbalance on safety accidents were evaluated through brain wave measurements. In addition, the effects of cumulative fatigue, physical vitality, autonomic nerve health, and autonomic balance were identified through pulse wave measurements. Data were acquired for 180 construction workers at various construction sites, and the workers were classified into three grades according to factors that affected safety accidents at construction sites. Then, the safety consciousness and safety commitment levels of workers corresponding to each grade of the influence factors were evaluated by conducting a questionnaire on safety consciousness and safety commitment. As a result, the characteristics of brain and pulse waves required to improve safety consciousness and safety commitment ability of workers at construction sites were explored.

## 1. Introduction

Industrial accidents refer to large and small accidents that can occur as a result of various production activities. Industrial accidents can lead not only to large-scale casualties, but also non-negligible economic losses. Korea has the highest mortality rate from industrial accidents among OECD countries, and the resulting economic loss is reported to be close to KRW 20 trillion [1]. The damages from accidents in the construction industry are the largest among the industrial accidents in various industries. It is reported that the number of injuries and fatalities of construction workers accounted for the highest percentages in all industrial accidents, at about 29% and 30%, respectively [2]. The construction industry is highly labor-intensive because the objectives need to be completed within a certain period of time, and it is extremely difficult and complex to manage a construction site where about 60 different types of work coexist. Therefore, the risk factors can increase exponentially at construction sites because the work situation is frequently changed and a substantial amount of mobile equipment is required for the work [3,4]. The risk of accidents for construction workers is further increasing because the work methods for construction sites are becoming more complex and diversified as the construction industry has grown [5]. Relatively more accident cases are being reported in the case of construction sites overseas within the construction industry due to unilateral contract documents related to safety, unsecured safety management costs, differences in safety culture, and large-scale and long-term work [6].

Unsafe behavior by workers is the biggest cause of accidents across industries, including the construction industry. The risk factors for accidents in the workplace increase as the proportion of workers who do not perform safe behavior increases [7,8]. Although the causes of unsafe behavior include defects or lack of knowledge, lack of work function, lack of consciousness of safety attitudes, and unique characteristics of each person, etc., the most likely causes are the negative consequences (inconvenience, time consumption, external pressure, etc.) caused by safe behavior [9]. Therefore, practical efforts to not only devise a physical environment that can reduce the risk but also correct workers’ unsafe behavior are required to prevent industrial accidents and achieve a safe state [10,11]. The elements required to control the unsafe behavior of workers are safety consciousness and safety commitment. Safety consciousness refers to knowledge of the work to be performed in order to ensure and sustain the safety of work at the site and an attitude to actively cope with this work. In other words, it refers to knowledge and a mindset of safety, which is closely related to the attitude of an individual worker toward safety [12]. On the other hand, safety commitment refers to the determination and mindset to continuously participate in safety consciousness. Therefore, it is affected by external factors such as the motivation of the members of the organization and the safety culture of the organization along with individual determination [13,14].

Research on various types of safety education to raise safety consciousness has been conducted as initial research to prevent accidents at construction sites (education for new workers, education for job changes, special safety and health education, regular education for workers, education for supervisors, etc.) [15]. Safety education refers to all education conducted for the purpose of preventing accidents at construction sites in advance and guiding actions to be taken to protect the safety and life of workers in the event of an unexpected accident. However, despite various types of safety education, there are limitations in that workers’ safety consciousness cannot be raised to a high level because the safety education is being provided meaninglessly and perfunctorily at the actual site [16]. Above all, research on safety commitment that can steadily maintain improved safety consciousness through education is not receiving much attention. Research on safety commitment has mainly been focused on improving commitment through external factors. New systems, laws, and regulations have been strengthened to prevent risk factors whenever there is a construction safety accident, and efforts have been made to establish a safety culture within organizations [5]. In addition, research on methods to continuously instill safety consciousness through communication by leaders at the site was also conducted [17,18].

The factors that have the greatest influence on safety commitment of workers, however, are not external factors but individual mental and health status. Psychological factors and physical factors such as physical fatigue and inadequate energy supply can cause unsafe behavior by interfering with individual safety commitment, and the incidence of safety accidents due to such unsafe behavior accounts for about 88% of all accidents [19]. It has been reported that many safety accidents occur due to the blood pressure, health status, and lifestyle of the elderly. In the case of the elderly, psychological factors such as low job satisfaction and attitude also contribute significantly to the incidence of safety accidents [20]. In addition, the risk of safety accidents increases due to cumulative physical fatigue and constant tension and stress if work is performed without sufficient rest. Health status, functioning level, and emotional stability due to stress should constantly be checked as well to prevent safety accidents [16]. In particular, research results show that workers who had previously experienced safety accidents had a high rate of recurrence of safety accidents due to negative emotions such as fear, tension, and stress about safety accidents stemming from trauma about the situation at that time. It was found that inexperienced workers were more likely to comply with safety rules than workers who had experienced safety accidents, even if the process was delayed [21]. Likewise, the health and mental states of workers have a significant influence on the formation of safety consciousness and safety commitment. Safety accidents do not occur sporadically but are caused by individual unsafe behavior and risk conditions accordantly; therefore, appropriate safety consciousness and safety commitments need to be formed by managing not only external risks but also personal health and mental status well in order to prevent safety accidents.

Therefore, the effect of the mental and health status of individuals at construction sites on safety consciousness and safety commitment was analyzed in this study. First, brain and pulse wave data from workers at construction sites were measured in order to evaluate individual mental and health status. A brain wave is an electrical signal measured using the electrical activity of electrodes attached to the surface of the scalp, which can appear in different forms depending on changes in dangerous or safe situations, control of emotions, and control of one’s internal and external body [22]. In addition, pulse waves are defined as all periodic phenomena that accompany the heart rate and are related to the autonomic nervous system; the autonomic nervous system adapts to environmental changes through the sympathetic and parasympathetic nerves. Only when the autonomic nervous system is healthy and balanced can it respond appropriately to stimuli from the external environment and increase the homeostasis of the human body by maintaining a constant state within the body [23]. Subsequently, the safety consciousness and safety commitment of workers were analyzed through a questionnaire survey and connected with brain and pulse wave data. As a result, the difference in safety consciousness and safety commitment according to brain and pulse waves was investigated, and the characteristics of brain and pulse waves required to improve safety consciousness and safety commitment ability were presented.

## 2. Theoretical Background

### 2.1. Analysis of Pulse Waves

Pulse waves are broadly defined as all periodic phenomena accompanying the heart rate and are highly related to the autonomic nervous system, which unconsciously controls the actions of organs, such as breathing and blood circulation, according to the integrated function of the hypothalamus. In this study, the health and mental state of workers according to pulse waves was determined by analyzing heart rate variability (HRV). Assessment of autonomic function by HRV has been rapidly developed worldwide due to papers published in 1996 by the European Society of Cardiology and the North American Society of Pacing and Electrophysiology [24]. HRV is expressed by converting the variation in the time interval between consecutive heartbeats into heart rate per minute, which can be used to characterize the activity, balance, and rhythm of the sympathetic and parasympathetic nerves constituting the autonomic nervous system [25,26,27]. Typically, risk factors to health are identified through power spectrum analysis [28,29,30].

**Figure 1:**
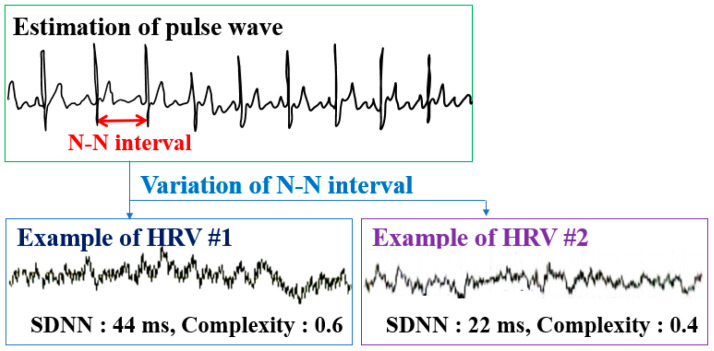
Determination of cumulative fatigue by heart rate variability (HRV) analysis.

The heart rate interval can be calculated by averaging the graph value of the HRV of an individual, and the heart rate can be determined by converting this into bmp units. The heart rate is an indicator of the level of arousal in the body, which increases with physical arousal and excitement [31]. Meanwhile, the standard deviation of the HRV graph is called the standard deviation of the N-N interval (SDNN), which reflects the level of the ability of autonomic nerves to adapt to external stress. Here, the N-N interval refers to the interval between peaks in HRV (Figure 1). The amplitude of the HRV waveform is greater when the SDNN is higher, which means that the width at which the heart rate interval changes is increased. In other words, the ability of the autonomic nervous system to adapt to changes in the external environment is higher when the SDNN is larger and the pattern is more complex, and conversely, it is less capable of responding to the external environment and coping with stress when the SDNN is the smaller and monotonous. In other words, the regulation ability of the vagus nerve, which acts as a pacemaker for heart rhythm, is lower when the SDNN is smaller (when the heart rate interval is more constant). As a result, the autonomic nervous system becomes unstable, and the ability to resist it is decreased even in small external stress situations [32,33]. In this study, cumulative fatigue was identified by analyzing the complexity of HRV (Figure 1), which represents the ability to cope with stress. When the complexity was about 0.6, it was classified as normal.

**Figure 2:**
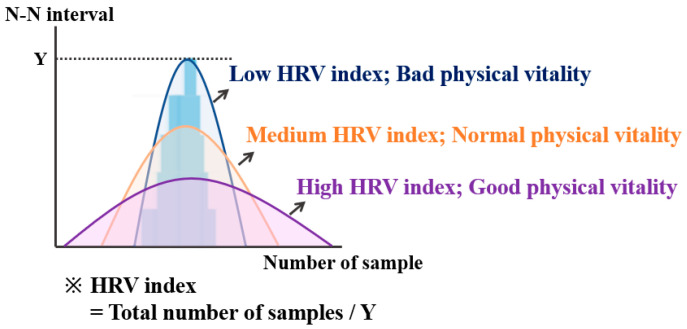
HRV-index and physical vitality evaluation with the probability distribution of HRV.

Cardiac function can be evaluated through geometric features by implementing a probability distribution from HRV. Normally, the probability distribution is low and has a broad width when healthy. On the other hand, autonomic nerve function is not healthy when the probability distribution is out of the normal range and has a high and narrow width [34]. The value obtained by comparing the shape of the probability distribution with the normal range is called the HRV-index. Then, the physical vitality of the worker can be evaluated according to the value of the HRV-index. In general, a larger HRV-index means a healthy person. In this study, it was judged that an HRV-index of 6.04 or less was not healthy (Figure 2).

**Figure 3:**
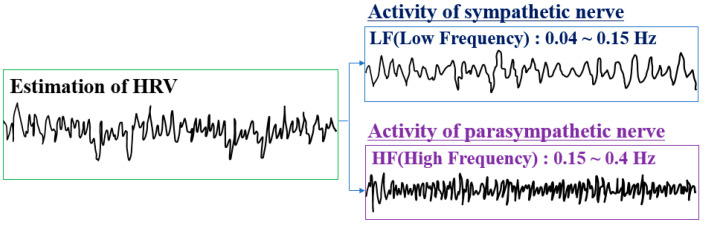
Determination of autonomic nervous system activation by power spectrum analysis of HRV.

The autonomic nervous system is mainly composed of the sympathetic and parasympathetic nervous systems. They are balanced by mutual antagonism. In general, the sympathetic nervous system is mainly active in aggressive and defensive stress situations, and the parasympathetic nervous system is more active in a comfortable and relaxed state. In a more detailed example, the sympathetic nervous system is first activated during the initial stress state, and this activated sympathetic nervous system accelerates heart rate, raises blood pressure and blood sugar, increases blood flow to voluntary muscles, causes perspiration, and decreases blood flow to internal organs. On the other hand, the parasympathetic nervous system is activated when the body is relaxed, leading to decreased heart rate and blood pressure, stimulation of saliva production, increased digestion, and sleep generation [35]. The sympathetic nerve is controlled at a low frequency and the parasympathetic nerve is controlled at a high frequency; therefore, the activity of the sympathetic nerve and the parasympathetic nerve can be grasped by analyzing the HRV with a power spectrum distribution (Figure 3).

The balance of the autonomic nerve can be grasped by deriving the relative activation ratio between the sympathetic and the parasympathetic [36]. When one of the sympathetic and parasympathetic nervous systems is excessively activated, the other side inhibits it and attempts to achieve balance through mutual regulation. The balance of the autonomic nerve can be used to determine whether these mutual regulation functions are working normally. If the activation is severely biased toward either the parasympathetic or the sympathetic for a long time, there is a risk that the nerves that are highly activated for a long time exhaust energy and eventually lose their function. During normal activities, the sympathetic nerve is slightly more active than the parasympathetic nerve, at a ratio of 6:4. In addition, the health of the autonomic nerve can be evaluated through the amplitudes of high frequency (i.e., parasympathetic nerve) and low frequency (i.e., sympathetic nerve) analyzed by the power spectrum distribution. In this study, when the total power was less than 5.16, it was judged as a risk level.

### 2.2. Analysis of Brain Waves

**Figure 4:**
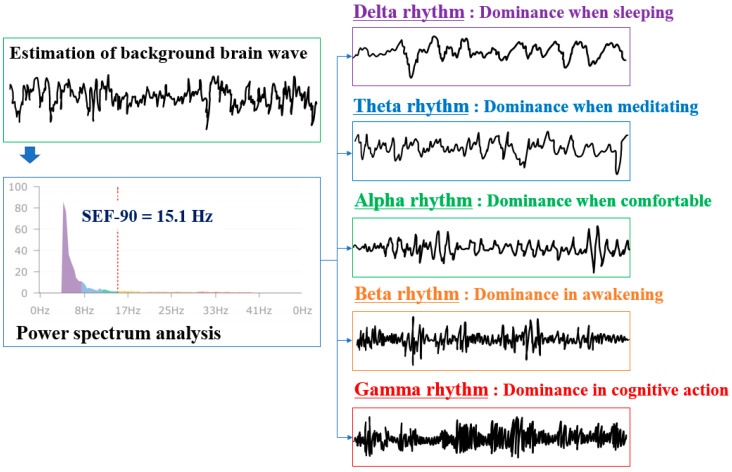
Power spectrum analysis of background brain wave and classification of brain wave rhythms for the degree of brain activity according to frequency.

The brain wave is a small signal from the electrical activity of neurons that compose the cerebral cortex measured at the scalp. The electrical activity of the brain reflected in brain waves is caused by neurons, glia cells, and the blood–brain barrier. Normally, changes in brain waves caused by glia cells and blood–brain barriers are small and slow, and changes in brain waves caused by neuronal activity are large, rapid, and diverse. Therefore, the brain waves have a waveform that vibrates in a very complex pattern [37]. Power spectrum analysis, as with pulse waves, is used to observe brain waves. Brain waves are classified into delta, theta, alpha, beta, and gamma rhythms through power spectrum analysis (Figure 4).

The thalamus is known to be the origin of the alpha rhythm, which becomes dominant when resting, according to visual stimuli [38,39]. In other words, the electrode power in the frequency band of the alpha rhythm becomes stronger than that of other rhythms. The electrical activity of cortical neurons measured when there is no external stimulation is called a background brain wave, and cognitive ability, brain function, brain disease, etc., can be determined through spectrum analysis of the alpha brain wave [40,41]. Theta and delta rhythms become dominant than alpha rhythms when becoming sleepy and less conscious. In contrast, beta and gamma rhythms become dominant when performing tasks or being overly aroused. The beta rhythm is further subdivided according to frequency, in which a faster beta rhythm becomes more dominant when the mental load is higher and in emotional tension, anxiety, and stress conditions [42,43]. If the power spectrum distribution of brain waves is biased toward higher frequencies compared to lower frequencies, it means that the mental workload level is extremely high for the work performed by workers [44,45,46]. The level of brain stress can be determined through the ratio of the intensity of the beta rhythm to the intensity of the alpha rhythm. The higher the ratio of the alpha rhythm, the less stress in the brain. In this study, a ratio of 1.2–1.5 was evaluated as having moderate brain stress.

Brain activity was evaluated through background brain waves. Background brain waves have a unique rhythm that occurs for anyone in a stable state, as shown in Figure 4, and the peak value is usually generates in the 5–13 Hz range. When brain activity is high, a peak value occurs around 10 Hz. As the level of brain activity decreases, the peak value frequency decreases and falls below 6.5 Hz in the dementia stage. In this study, when spectral edge frequency (SEF)-90 was 11.7~19.5 Hz, it was concluded to have normal brain activity.

**Figure 5:**
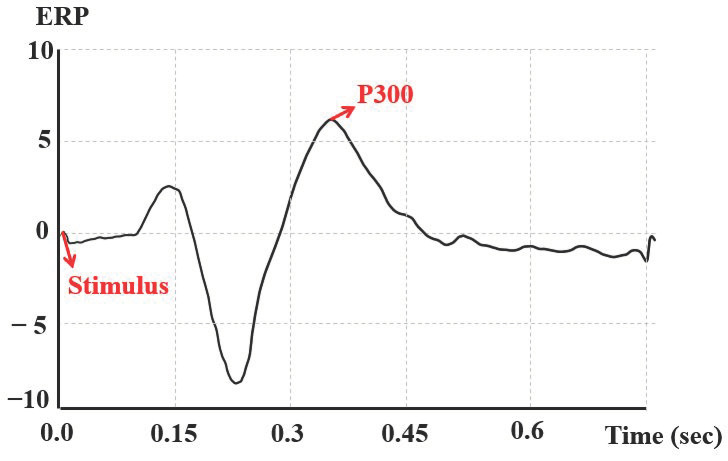
Determination of P300 through event-related potential (ERP) for evaluating concentration.

The concentration of workers was also identified using brain waves. The brain wave responses related to attention are measured through the active oddball task. This is an experiment in which the response and brain waves of research subjects are measured when a stimulus is presented. Here, the brain potential generated for a specific stimulus is identified through a potential difference, which is called an event-related potential (ERP). In this study, the degree of concentration was determined by identifying the peak that occurs after a certain period of time after presenting the auditory stimulus, called the brain potential (brainstem auditory evoked potential, BAEP) [47]. The positive peak that is shown around 300 ms after the presentation of the stimulus is called P300 and reflects selective attention, stimulus cognition, memory search, and relief of a sense of uncertainty [48]. Attention, memory, and cognitive ability tend to be higher when the amplitude of P300 is increased and when it is shown earlier [49]. In this study, the degree of concentration was evaluated by measuring the P300 value for repetitive stimulation, as shown in Figure 5. A P300 value of five was standard for evaluating the high or low degree of concentration.

Lastly, higher-order cognitive function can be tested with the discrimination task. The pattern presented on the screen is first stored in short-term memory, and the ability to determine whether the pattern matches the previously remembered pattern when the pattern is presented subsequently is evaluated through reasoning and comparison [50]. In general, higher-order cognitive functions, in which perception, attention, memory, reasoning, and determination functions are efficiently associated, are evaluated by the induced gamma response [43]. In addition, the activity and balance of the left and right brain can be identified with the percentage of gamma activity of the left and right brain, because gamma power is remarkably activated when higher-order cognitive function is activated. Problem-solving tendencies of logical, analytical, linguistic, mathematical, and sequential information processing methods more familiar when the left brain is relatively more active, whereas the problem-solving tendencies of similar, intuitive, non-verbal, spatial, and holistic information processing methods are more familiar when the right brain is relatively more active. In addition, it has been reported that the right brain is abnormally activated in the frontal lobe in the case of depression, and training to balance the brain wave activity of the left and right brain is being researched as a remedy [51]. To determine the imbalance of the left and right brain, the background brain wave was classified through the power spectrum, as shown in Figure 4, and the gamma power of the left and right brain was measured and compared.

## 3. Research Method

### 3.1. Research Subjects and Bio-Signal Measurement Method

First, the research subjects were selected to acquire brain and pulse wave data in order to analyze the difference in safety consciousness and safety commitment according to brain and pulse waves among construction workers. The brain and pulse wave measurement period was set at five months in consideration of hot and cool weather in order to minimize the influence of conditions that can affect brain and pulse waves, and it was distributed in balance for each region, including Ulsan, Incheon, Daejeon, and Busan. In addition, five construction companies were contacted to recruit workers from various environments regarding the construction scale, construction type, construction period, and construction cost. As a result, 180 of the 182 construction site workers who agreed to the purpose of the research were recruited, and data for each job were measured from June to the end of October, 2020. The age, gender, and career information for the 180 workers is shown in Table 1.

The brain and pulse waves were measured using a portable autonomic balance function diagnostic device. This tool is a class 2 medical equipment measurement device that transmits the measured data through Bluetooth by measuring the signal of each channel through the two channels of electroencephalogram (EEG) electrodes and by measuring the light reflection from the peripheral blood vessels and the pulse wave signal from the blood flow through the photoplethysmogram (PPG) [52]. Two-channel electrodes were used to measure brain waves in the left and right brains. A headset for measuring the brain activity of brain waves was worn on the forehead for brain wave measurement, and a sensor for measuring the brain stress of pulse waves was attached to the earlobe for pulse wave measurement [53]. Brain waves were measured for one minute per every measurement while the subject was seated in a comfortable chair in a stable state with eyes closed. It was necessary to maintain stability during measurement; therefore, long-term measurement was avoided, and measurement was performed for one minute. The subject was cautioned not to perform tasks that artificially induce mental loads such as mental arithmetic or intensive meditation during the measurement. In addition, the pulse wave was measured in a non-invasive manner for one minute per every measurement by using a pulse wave meter while the subject was seated in a comfortable chair in a stable state with eyes closed. The hand attached to the sensor was placed on a table at the height of the heart and was maintained in a comfortably relaxed state, and nail polish or foreign substances on the fingernails attached to the pulse wave electrode were removed. In addition, factors that compressed the arm or fingers, such as a tight top, disposable band, or rubber band, were also removed. The subjects were cautioned to refrain from taking deep or abdominal breaths during the pulse wave measurement.

Variables that can affect safety accidents at construction sites were extracted through the measured results of brain and pulse waves. First, the level of brain stress and brain activity was selected by measuring the level of relaxation of the brain through brain waves [39]. Most workers work without being properly conscious of their fatigued physical state or of negative emotional states such as stress, tension, and fear. Higher brain stress was shown in such a mentally stressed state of mental anxiety, tension, nervousness, or excessive arousal, compared to the standard range, and the risk of an accident may vary when the body reacts to such stress [16]. In addition, the left and right brain balance and concentration were determined through brain waves in order to determine the level of concentration at the construction site. Brain stress, brain activity, and concentration were classified into three levels, low, moderate, and high, and the left and right brain imbalance was analyzed by dividing it into left-brain type, whole-brain type, and right-brain type. Among the pulse waves, cumulative fatigue and physical vitality, which had a serious effect on safety accidents, were measured, and autonomic balance and autonomic nerve health were evaluated. Cumulative fatigue was divided into normal, caution, and dangerous, and physical vitality was divided into low, moderate, and high. Cumulative fatigue refers to the level of fatigue accumulated physically and mentally due to repeated stress [54]. Such fatigue and stress cause the human body to become mentally and physically unhealthy by disturbing the activities of the autonomic nervous system [55]. In addition, a stressful situation results in an increase in blood pressure and pulse rate as well as an increase in cortisol due to the endocrine system by inducing a reaction of the autonomic nervous system, which may affect brain waves. Therefore, the autonomic balance was divided into dominant sympathetic nervous system, balanced, and dominant parasympathetic nervous system, and the autonomic nerve health was divided into at risk, good, and healthy for evaluation. The left and right brain imbalance and autonomic balance were divided into three groups; therefore, other factors were also divided into three levels for ease of statistics and analysis.

Variables derived from brain and pulse wave measurements were values obtained from standardized statistical analysis results [56]. The variables of the research subject classified according to brain and pulse wave types are as shown in Table 2 and Table 3, respectively.

### 3.2. Safety Consciousness and Safety Commitment Determination through a Survey

As mentioned above, safety consciousness is an attitude of actively coping with individual safety, which is knowledge of what needs to be performed to ensure and sustain the safety of work at the site. On the other hand, safety commitment refers to an individual’s mental attitude in which the determination and belief to continuously participate in behavior are formed. In other words, safety commitment can lead to continuous behaviors to prevent safety accidents. A questionnaire survey was conducted in this study to determine the level of safety consciousness and safety commitment of construction workers by measuring brain and pulse waves. This was to analyze the relationship between brain wave variables (brain stress, brain activity, concentration, left and right brain imbalance) and pulse wave variables (cumulative fatigue, physical vitality, autonomic balance, autonomic nerve health) that affect safety accidents according to the safety consciousness and safety commitment of construction workers, because the occurrence of a safety accident can be determined by the level of such workers’ safety consciousness and safety commitment.

The questionnaire on safety consciousness and safety commitment was revised and supplemented to suit the construction site workers, who were the subjects of this study, based on the research of Ryu and Kim [57,58]. The questionnaire for safety consciousness and safety commitment as well as the Cronbach’s α values are shown in Table 4. The Cronbach’s α value verifies whether there is no problem even when a variable is generated by taking the average of whether there is consistency between questionnaire items, which was derived through the reliability analysis of the items. The Cronbach α value ranges from 0 to 1, and a value of 0.8 or more means that the questionnaire items are well-formed with correlation [59].

## 4. Brain Waves and Brain Wave Analysis Results

The influencing factors of brain and pulse waves were classified into three grades, respectively, and the level of safety consciousness and safety commitment of workers corresponding to each grade was evaluated to analyze the difference in safety consciousness and safety commitment according to the brain and pulse waves of workers at construction sites. The derived safety consciousness and safety commitment scores were obtained as a standardized score of 0–5 points according to the grade of the influencing factors that can affect safety accidents at construction sites. The representativity of the statistical probability distribution of a normal distribution was examined through the bell curve of the histogram and the linearity of the Normal Q-Q plot, and statistically significant differences in the results were examined through whether the significance goodness-of-fit level was 0.05 or higher [60]. An *F*-test and an analysis of variance (ANOVA), was performed to determine the level of significance [61]. Then, a post hoc analysis following the Scheffe method used when equal variance was assumed was performed if statistically significant differences were found between groups [62].

### 4.1. Analysis of Difference in Safety Consciousness and Safety Commitment According to Brain Waves of Workers at Construction Sites

**Figure 6:**
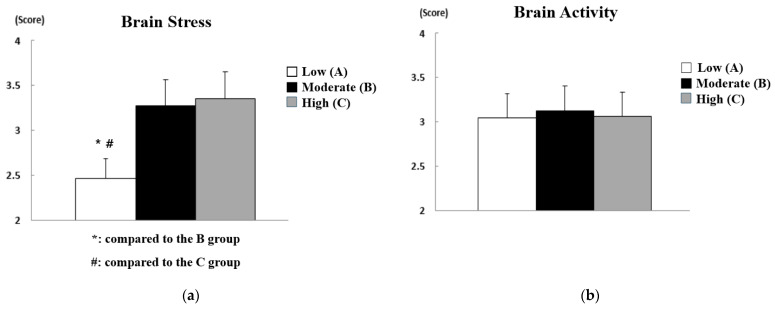

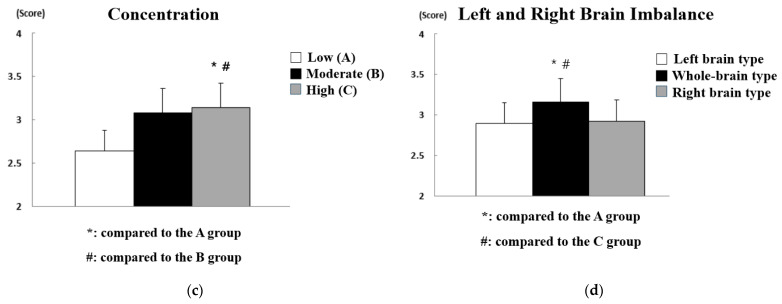
Analysis of differences in safety consciousness according to brain wave data: (**a**) effect of brain stress; (**b**) effect of brain activity; (**c**) effect of concentration; (**d**) effect of left and right brain imbalance.

The results of analyzing the difference in safety consciousness according to the brain waves of workers at construction sites are shown in Figure 6, and the statistical analysis results are summarized in Table 5. In Figure 6, the *y*-axis represents the score for safety consciousness, and the *x*-axis represents the three-level grade of influencing factors that can affect safety accidents at construction sites. In other words, they show the average score for the safety consciousness of workers corresponding to each grade of the influence factors.

As a result of statistical analysis of the safety consciousness questionnaire, statistically significant differences were found in brain stress (*p* < 0.01), concentration (*p* < 0.01), and left and right brain imbalance (*p* < 0.05). On the other hand, no statistically significant difference was not found in the level of brain activity, which means that major and minor brain activity did not significantly affect safety consciousness. Therefore, post hoc analysis was performed only on the factors of brain stress, concentration, and left and right brain imbalance. First, workers at construction sites with low brain stress were observed to have a significantly lower level of safety consciousness than workers at construction sites with moderate or high brain stress. One might think that it is difficult to be conscious of safety due to tension and anxiety in the brain when there is a lot of brain stress, but it was found that it instead helped workers constantly keep in mind and pay attention to safety. In addition, of course, workers at construction sites with high concentration were evaluated to have higher safety consciousness than workers at construction sites with low or moderate concentration, and whole-brain type construction site workers were evaluated to have higher safety consciousness than left-brain type or right-brain type construction site workers. The * and # marks in Figure 6 indicate that there is a significant difference compared to other groups.

**Figure 7:**
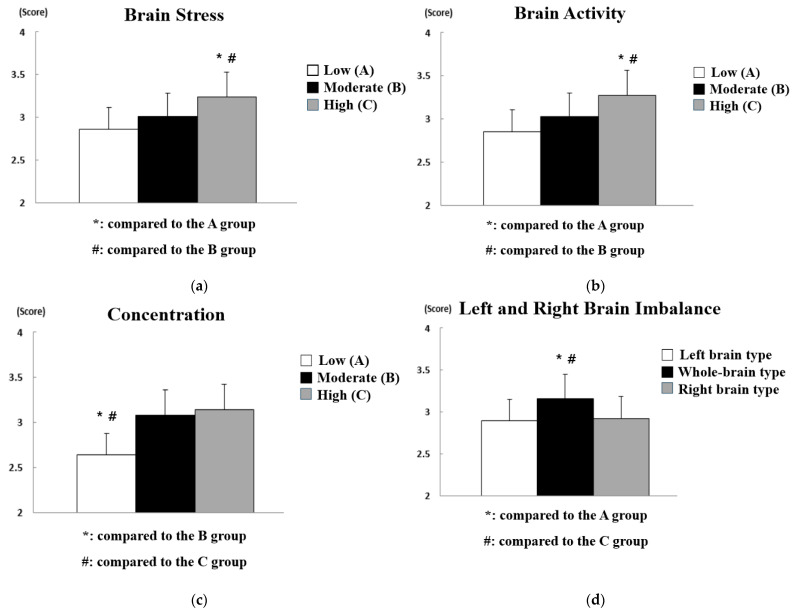
Analysis of differences in safety commitment according to brain wave data: (**a**) effect of brain stress; (**b**) effect of brain activity; (**c**) effect of concentration; (**d**) effect of left and right brain imbalance.

The results of the analysis of differences in safety commitment according to the brain waves of workers at construction sites are shown in Figure 7, and the statistical analysis results are summarized in Table 6.

As a result of statistical analysis of the safety commitment questionnaire result, all influencing factors (i.e., brain stress (*p* < 0.01), brain activity (*p* < 0.01), concentration (*p* < 0.01), and left and right brain imbalance (*p* < 0.05)) showed a statistically significant difference. Therefore, post hoc analysis was performed on all influencing factors. First, as in the safety consciousness analysis results, it was found that workers at construction sites with high brain stress were observed to have a significantly higher level of safety commitment than workers at construction sites with low or moderate brain stress. On the other hand, contrary to the results of safety consciousness analysis on the level of brain activity, workers with high brain activity showed higher levels of safety commitment. In other words, it can be concluded that the level of brain activity affects the level of maintaining it rather than knowledge and attitudes about safety. In the areas of concentration and left–right brain imbalance, it was found that whole-brain-type workers at construction sites with high concentration showed higher safety commitment and safety consciousness.

### 4.2. Analysis of Difference in Safety Consciousness and Safety Commitment According to Pulse Waves of Workers at Construction Sites

**Figure 8:**
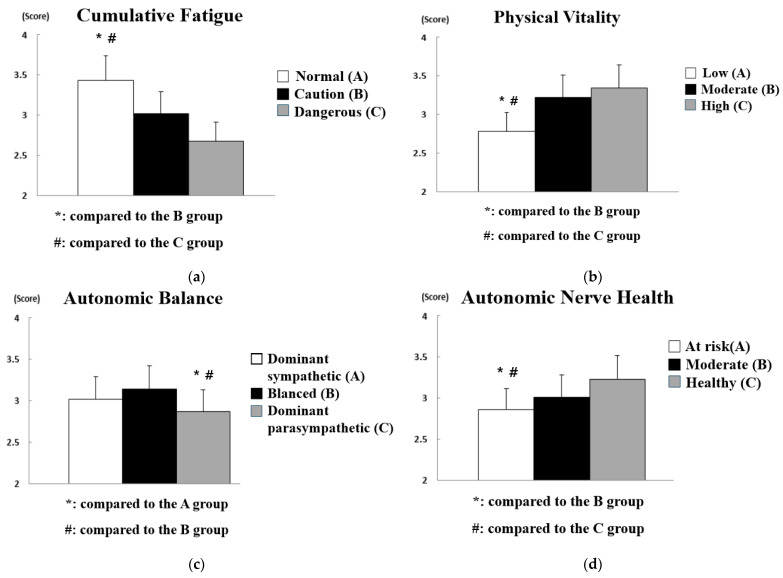
Analysis of differences in safety consciousness according to pulse wave data: (**a**) effect of cumulative fatigue; (**b**) effect of physical vitality; (**c**) effect of autonomic balance; (**d**) effect of autonomic nerve health.

The results of analyzing the difference in safety consciousness according to the pulse waves of workers at construction sites are shown in Figure 8, and the statistical analysis results are summarized in Table 7.

As a result of analyzing the difference in safety consciousness according to the pulse waves of workers at construction sites, statistically significant differences were found in all influencing factors (i.e., cumulative fatigue (*p* < 0.01), physical vitality (*p* < 0.05), autonomic balance (*p* < 0.01), and autonomic nerve health (*p* < 0.05)). First, it was found that the construction site workers who were at risk due to the high cumulative fatigue level had a markedly low safety consciousness compared to the construction site workers whose cumulative fatigue was at caution level or normal. In addition, construction site workers with low physical vitality had much lower safety consciousness than construction site workers with moderate or high physical vitality. In other words, fatigue and physical vitality caused by constant physical labor at the construction site need to be continuously managed through appropriate rest and various on-site programs. In particular, the risk of accidents will increase exponentially at high-risk construction sites because mental fatigue is likely to increase further. In terms of autonomic balance, it was found that workers at construction sites with a dominant parasympathetic nervous system or a balanced autonomic nervous system had higher safety consciousness than workers at construction sites with a dominant sympathetic nervous system. As described above, the sympathetic nervous system is mainly active in aggressive and defensive stress situations, and the parasympathetic nervous system is more active in a comfortable and relaxed state. In other words, workers at construction sites with a dominant parasympathetic nervous system will likely be in a state of lack of safety consideration because it is easy to maintain a state of reduced tension and attention to safety. Finally, the autonomic nerve health level refers to the activity of the sympathetic and parasympathetic nervous systems. The higher the autonomic nerve health, the younger and healthier the worker. However, autonomic nerve health may be high even if only one of the sympathetic and parasympathetic nervous systems is highly activated due to the low autonomic balance. Safety consciousness was evaluated as higher in construction site workers with moderate or healthy autonomic health than in those with autonomic health at risk.

**Figure 9:**
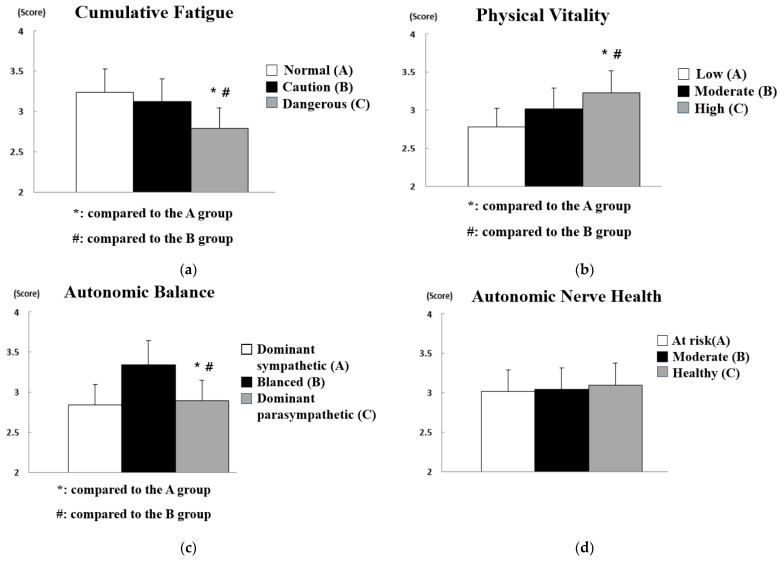
Analysis of differences in safety commitment according to pulse wave data: (**a**) effect of cumulative fatigue; (**b**) effect of physical vitality; (**c**) effect of autonomic balance; (**d**) effect of autonomic nerve health.

The results of analyzing the difference in safety commitment according to the pulse waves of workers at construction sites are shown in Figure 9, and the statistical analysis results are summarized in Table 8.

In the analysis results for the difference in safety commitment according to the pulse waves of workers at construction sites, statistically significant differences were found only in cumulative fatigue (*p* < 0.01), physical vitality (*p* < 0.01), and autonomic balance (*p* < 0.01), while no significant difference was found in autonomic nerve health. In other words, the activity of the sympathetic or parasympathetic nervous system only affects the attitude toward safety and does not have a significant effect on maintaining the attitude. First, it was found that the safety commitment of construction site workers with high cumulative fatigue was significantly lowered, and it was observed that construction site workers with high physical vitality had a higher safety commitment than construction site workers with low or normal physical vitality. It can thus be concluded that the management of fatigue and vitality of workers is essential in terms of safety consciousness and safety commitment. In addition, construction site workers with a dominant sympathetic nervous system or a balanced autonomic nervous system showed higher safety commitment than those with a dominant parasympathetic nervous system. This suggests that workers at construction sites with a dominant parasympathetic nervous system need special attention in terms of safety consciousness and safety commitment.

## 5. Discussion

In this study, the vulnerability and risk factors for safety consciousness and safety commitment in construction sites were determined through the measurement of brain and pulse waves. As a result of brain wave measurements, it was found that brain stress, concentration, and left and right brain imbalance play an important role in safety consciousness, and it was evaluated that the degree of brain activity additionally affects safety commitment. In the pulse wave measurement results, cumulative fatigue, physical vitality, and autonomic balance play an important role in safety commitment, and autonomic nerve health was added as an influencing factor for safety consciousness.

Health factors of the brain and body determined by brain and pulse waves affect safety consciousness and safety commitment; therefore, safety accidents in construction sites can be exponentially reduced by managing the brain and body health of workers. The workers evaluated in the risk level of safety consciousness or safety commitment can be excluded from high-risk work or have appropriate rest by measuring brain and pulse waves in real time. In addition, it is possible to identify and prepare for emergencies in advance, which is a very effective accident prevention countermeasure at construction sites that have many dangerous works and large site sites. Such a system can be implemented by big data and machine learning technology that acquires and analyzes brain waves and pulse waves in real time. However, more precise and reliable factor analysis should be preceded by establishing a database for brain and pulse waves of more workers.

In addition, safety consciousness and safety commitment at construction sites can be increased, and the occurrence of safety accidents can be drastically decreased by introducing a program to manage and train brain and pulse waves. The first is training programs to control brain waves. Brain waves vary according to the active state of the brain, and specific brain waves can be controlled when feedback is received through various training regimes for the brain [63]. Humans have brain plasticity in which the structure and function of the brain changes after birth according to a given environment, experience, needs, and demands; therefore, brain wave training is a scientific and effective method that improves the plasticity of the brain by using brain waves and activates the brain by itself through the reorganization or re-composition of nervous tissues in the brain and networks [64]. Likewise, the safety consciousness and safety commitment of workers regarding safety accidents can be improved by stabilizing brain waves and improving brain plasticity through brain wave training.

Stress management training for the brain also greatly helps improve safety consciousness and safety commitment. Unsafe behavior in response to accidents that may occur at the site is caused by reactions to stress [13]. In particular, stress responses such as depression and anxiety were found to have a negative effect on the safety behavior of workers at construction sites [65]. These psychological factors as well as physical factors of physical fatigue and inadequate energy supply can cause unsafe behavior in individual people, and the incidence of safety accidents due to unsafe behavior accounts for about 88% of all accidents [19]. Therefore, stress management training to control stress at a certain level, such as an appropriate rest and healing program, will be required to improve safety consciousness and safety commitment.

Training to improve the ability to regulate the autonomic nervous system can also be applied. The autonomic nervous system adapts to environmental changes by controlling sensory motor, visceral, and endocrine functions through the sympathetic and parasympathetic nerves. This ability to regulate the autonomic nervous system can maintain its balance harmoniously by appropriately responding to stimuli from the external environment through training and improve the homeostasis of the human body by maintaining a constant state [63]. In other words, the safety consciousness and safety commitment of workers can be raised by increasing the balance and health of the autonomic nerve through enhancement of the autonomic function.

However, in order to introduce management and training programs for the brain and pulse waves, further research will be needed on what effect it will have. Based on the results of this study, which derived risk factors on safety accidents caused by workers through brain and pulse wave measurements, a study to measure the degree of safety commitment and safety awareness improvement through management and training should be conducted in the future.

## 6. Conclusions

The purpose of this study was to collect basic data for developing technologies to prevent safety accidents at construction sites in the future through brain and pulse wave measurements by analyzing the differences in safety consciousness and safety commitment according to analysis of the brain and pulse waves of workers at construction sites. Therefore, data were acquired and analyzed by measuring the brain and pulse waves of 180 construction workers at various construction sites, and the workers were classified into three grades according to factors that affected safety accidents at construction sites. Subsequently, the safety consciousness and safety commitment levels of workers corresponding to each grade of the influence factors were evaluated by conducting a questionnaire on safety consciousness and safety commitment. The results of the research can be summarized and analyzed as follows.

1. First, as a result of analyzing the difference in the safety consciousness of workers at construction sites according to brain waves, the safety consciousness was higher when brain stress and concentration were higher. An appropriate level of stress is an essential factor in human growth that can play not only a dysfunctional role but also a functional role. In other words, it was found that a certain level of brain stress is required to have the safety consciousness to cope with construction site workers’ safety accidents. However, it was observed that the level of brain activity did not significantly affect the safety consciousness of workers at construction sites. On the other hand, more whole-brained workers had higher safety consciousness. The safety consciousness can be improved when the left and right brains interact without being biased to either side and form experiences through integrated processing because the two hemispheres of the brain share stimuli with each other through the corpus callosum and work as an integrated whole.
2. As in safety consciousness, in terms of the safety commitment of workers at construction sites, according to brain waves, the safety commitment of whole-brain-type workers with high brain stress and concentration was observed to be higher. In particular, it was found that workers showed better safety commitment when the level of brain activity was higher, unlike in the case of safety consciousness. In other words, not only a certain level of brain stress but also the level of brain activity needs to be high to continuously maintain the behavior of preventing safety accidents within the organization through commitment. In addition, workers with low levels of brain activity work without correctly recognizing their fatigued physical state or negative emotional states such as stress, tension, and fear. If this condition persists, it may negatively affect the brain, leading to a decrease in brain stress and concentration.
3. According to the pulse wave, both safety consciousness and safety commitment were high when cumulative fatigue was normal and physical vitality was normal or high. In addition, both safety consciousness and safety commitment were high when the autonomic nerve was balanced. However, the health of the autonomic nerve was found to have a greater impact on safety consciousness than on safety commitment. If the stress due to internal and environmental changes persists in the body, humans cannot adequately respond to external stimuli due to cumulative fatigue and cannot maintain safety consciousness because the heart rate decreases due to the decreased ability to control stress. This phenomenon may be clearly observed as the autonomic nerve activation decreases.

## Figures and Tables

**Table 1:** Information of workers who participated in experiments.

Variable	Information	*n* ^1^	Ratio
Age	Aged in their 20s	8	4.44%
	Aged in their 30s	29	16.11%
	Aged in their 40s	45	25.00%
	Aged over 50	98	54.44%
Gender	Male	172	95.56%
	Female	8	4.44%
Career	Less than 5 years	28	15.56%
	5–10 years	39	21.67%
	10–15 years	53	29.44%
	More than 15 years	60	33.33%

^1^ Number of subjects corresponding to each variable.

**Table 2:** Variables of the research subject classified by brain wave data.

Variable	Grade	*n* ^1^
Brain stress	Low (A)	52
	Moderate (B)	83
	High (C)	45
Brain activity	Low (A)	34
	Moderate (B)	108
	High (C)	38
Concentration	Low (A)	46
	Moderate (B)	94
	High (C)	40
Left and right brain imbalance	Left-brain type (A)	42
	Whole-brain type (B)	98
	Right-brain type (C)	40

^1^ Number of subjects corresponding to each variable.

**Table 3:** Variables of the research subject classified by pulse wave data.

Variable	Grade	*n* ^1^
Cumulative fatigue	Normal (A)	45
	Caution (B)	82
	Dangerous (C)	53
Physical vitality	Low (A)	40
	Moderate (B)	99
	High (C)	41
Autonomic balance	Dominant sympathetic nervous system (A)	38
	Balanced (B)	101
	Dominant parasympathetic nervous system (C)	41
Autonomic nerve health	At risk (A)	43
	Moderate (B)	97
	Healthy (C)	40

^1^ Number of subjects corresponding to each variable.

**Table 4:** Questionnaire for determining safety consciousness and safety commitment of workers.

Item	No.	Questionnaire	Cronbach’s α
Safety consciousness	1	I think the safety rules of the workplace must be complied with.	0.863
	2	I think accidents can happen at any time during work.	
	3	I think safety needs to be taught before working.	
	4	I have a fear of accidents.	
	5	I think safety comes first over progress of process.	
	6	Safety accidents can be prevented only when all workers are careful.	
	7	I’m more conscious of safety than other workers.	
Safety commitment	1	I know the methods to cope with safety accidents.	0.894
	2	I know the safety regulations and procedures of the workplace.	
	3	I’m familiar with the risk factors of accidents on work machines and facilities.	
	4	I know the protective equipment required for the job.	
	5	I have a steady interest in preventing safety accidents.	
	6	I know the safety hazards in the workplace.	
	7	A moment of inattention can lead to an accident.	
	8	Minor carelessness can lead to safety accidents.	
	9	Safety accidents can happen at any time.	
	10	It is everyone’s duty to comply with safety regulations and procedures.	
	11	Safety is connected to my life.	
	12	There is a high possibility of a safety accident if safety regulations and procedures are not complied with.	
	13	I comply to the procedure for any job.	
	14	I do not enter hazardous areas.	
	15	I do not ignore even the slightest risk factors.	
	16	I’m not overconfident in my skills.	
	17	It’s more important to work safely than to get jobs done quickly.	

**Table 5:** Statistical analysis results for differences in safety consciousness according to brain wave data.

Variable	Grade	*n*	M ^1^	SD ^2^	*p*-Value	Post Hoc Analysis
Brain stress	Low (A)	52	2.46	0.874	0.004	A < B A < C
	Moderate (B)	83	3.27	0.563		
	High (C)	45	3.35	0.662		
Brain activity	Low (A)	34	3.04	0.598	0.104	-
	Moderate (B)	108	3.12	0.646		
	High (C)	38	3.06	0.642		
Concentration	Low (A)	46	2.78	0.732	0.002	A < C B < C
	Moderate (B)	94	2.80	0.578		
	High (C)	40	3.45	0.689		
Left and right brain imbalance	Left-brain type (A)	42	2.90	0.650	0.024	A < B C < B
	Whole-brain type (B)	98	3.24	0.568		
	Right-brain type (C)	40	2.97	0.683		

^1^ Mean value of score for safety consciousness; ^2^ standard deviation of score for safety consciousness.

**Table 6:** Statistical analysis results for differences in safety commitment according to brain wave data.

Variable	Grade	*n*	M ^1^	SD ^2^	*p*-Value	Post Hoc Analysis
Brain stress	Low (A)	52	2.86	0.835	0.005	A < C B < C
	Moderate (B)	83	3.01	0.535		
	High (C)	45	3.24	0.658		
Brain activity	Low (A)	34	2.85	0.578	0.006	A < C B < C
	Moderate (B)	108	3.03	0.636		
	High (C)	38	3.27	0.637		
Concentration	Low (A)	46	2.64	0.732	0.003	A < B A < C
	Moderate (B)	94	3.08	0.578		
	High (C)	40	3.14	0.689		
Left and right brain imbalance	Left-brain type (A)	42	2.89	0.783	0.032	A < B C < B
	Whole-brain type (B)	98	3.16	0.683		
	Right-brain type (C)	40	2.92	0.649		

^1^ Mean value of score for safety commitment; ^2^ standard deviation of scores for safety commitment.

**Table 7:** Statistical analysis results for differences in safety consciousness according to pulse wave data.

Variable	Grade	*n*	M ^1^	SD ^2^	*p*-Value	Post Hoc Analysis
Cumulative Fatigue	Normal (A)	45	3.43	0.674	0.001	B < A C < A
	Caution (B)	82	3.02	0.536		
	Dangerous (C)	53	2.67	0.635		
Physical vitality	Low (A)	40	2.78	0.542	0.027	A < B A < C
	Moderate (B)	99	3.22	0.629		
	High (C)	41	3.34	0.625		
Autonomic balance	Dominant sympathetic (A)	38	3.02	0.742	0.007	C < A C < B
	Balanced (B)	101	3.14	0.526		
	Dominant parasympathetic (C)	41	2.87	0.627		
Autonomic nerve health	At risk (A)	43	2.86	0.638	0.029	A < B A < C
	Moderate (B)	97	3.01	0.537		
	Healthy (C)	40	3.23	0.627		

^1^ Mean value of score for safety consciousness; ^2^ standard deviation of scores for safety consciousness.

**Table 8:** Statistical analysis results for differences in safety commitment according to pulse wave data.

Variable	Grade	*n*	M ^1^	SD ^2^	*p*-Value	Post Hoc Analysis
Cumulative Fatigue	Normal (A)	45	3.24	0.455	0.003	C < A C < B
	Caution (B)	82	3.12	0.532		
	Dangerous (C)	53	2.79	0.636		
Physical vitality	Low (A)	40	2.78	0.578	0.006	A < C B < C
	Moderate (B)	99	3.02	0.628		
	High (C)	41	3.23	0.638		
Autonomic balance	Dominant sympathetic (A)	38	2.84	0.683	0.001	C < A C < B
	Balanced (B)	101	3.34	0.527		
	Dominant parasympathetic (C)	41	2.89	0.680		
Autonomic nerve health	At risk (A)	43	3.02	0.630	0.085	-
	Moderate (B)	97	3.04	0.563		
	Healthy (C)	40	3.10	0.620		

^1^ Mean value of score for safety commitment; ^2^ standard deviation of scores for safety commitment.

## Data Availability

The data presented in this study are available on request from the corresponding author. The data are not publicly available due to privacy.
